# Giant Leiomyosarcoma Arising in Posterior Thigh: Management of a Rare Case

**DOI:** 10.7759/cureus.10146

**Published:** 2020-08-30

**Authors:** Tehlil Rizwan, Jawad Ahmed, Fahad H Shaikh, Farheen Malik, Shuah Ullah

**Affiliations:** 1 Internal Medicine, Dow University of Health Sciences, Karachi, PAK; 2 General Surgery/Urology, Sindh Institute of Urology and Transplantation, Karachi, PAK

**Keywords:** leiomyosarcoma, sarcoma, smooth muscle malignancy, thigh, compartmental excision, leiomyosarcoma of somatic soft tissues

## Abstract

Leiomyosarcoma, primarily a tumor of smooth muscle origin, frequently originates from the uterus, retroperitoneum, and intra-abdominal region. Rarely, the tumor may arise from the conjunctiva, inferior vena cava, or oral cavity. Here we report a case of a 65-year-old male patient who presented with a swelling in the posterior thigh for six months. The swelling was progressively increasing in size for the last two months. Examination of thigh showed a swelling of 20×30 cm in size, which was firm, non-compressible, immobile, and not transilluminating. CT scan showed no metastasis in the liver, lung, or bone. The histopathology report showed poorly differentiated leiomyosarcoma involving the muscles of the posterior compartment of the left thigh. The tumor was resected, and the patient was referred to rehabilitation clinic. Early diagnosis of such cases is essential to improve the outcome in patients as these tumors can metastasize early.

## Introduction

Leiomyosarcoma is a malignant tumor of the smooth muscle connective tissue. The National Organization for Rare Disorders (NORD) enlists leiomyosarcoma as a rare malignancy [1]. Soft tissue sarcomas account for 0.7% of all malignancies, and out of these, leiomyosarcomas make only 5%-10% of the total cases. In the United States, the incidence of leiomyosarcoma is less than 1 in 200,000 people [2]. Leiomyosarcoma usually arises from the uterus, retroperitoneum, and intra-abdominal region [3]. Due to its rarity, the exact etiology and risk factors for leiomyosarcoma are not clearly understood [1]. Initially, the patient is asymptomatic, and diagnosis is usually made in later stages when the disease has metastasized to sensitive areas such as the skin, liver, lung, bone, brain, and soft tissue [4].

Rare cases of leiomyosarcoma arising in the oral cavity, conjunctiva, and inferior vena cava have been reported in the literature [5-7]. An extensive and thorough literature search revealed only few case of leiomyosarcoma arising from lower extremity muscles [8,9]. To the best of our knowledge, this is the first reported case of leiomyosarcoma originating from thigh muscles in Pakistan. The rarity of the tumor, along with its unusual site of origin, makes this a compelling case to report. Here we present a case of a 65-year-old male who presented with the complaint of a swelling on posterior thigh, which was non-tender but progressively increased in size.

## Case presentation

A 65-year-old male, with no known comorbidities, presented to the outpatient department (OPD) of Civil Hospital Karachi with a swelling on the posterior side of the left upper thigh for six months. The swelling had been progressively increasing in size and interfered with the mobility of the patient. History for anorexia, weight loss, and constipation was positive. The patient was a smoker and had been smoking (one pack/week) for the last 15 years. Family history was negative for any chronic or malignant disease. On general physical examination, the patient was afebrile, pale, and lethargic. There were no palpable lymph nodes, and only solitary swelling was noted on the left thigh. All reflexes were intact, and the patient had a Glasgow Coma Scale (GCS) score of 15/15. Respiratory, gastrointestinal, cardiovascular, and all other systemic examinations were unremarkable.

**Figure 1:**
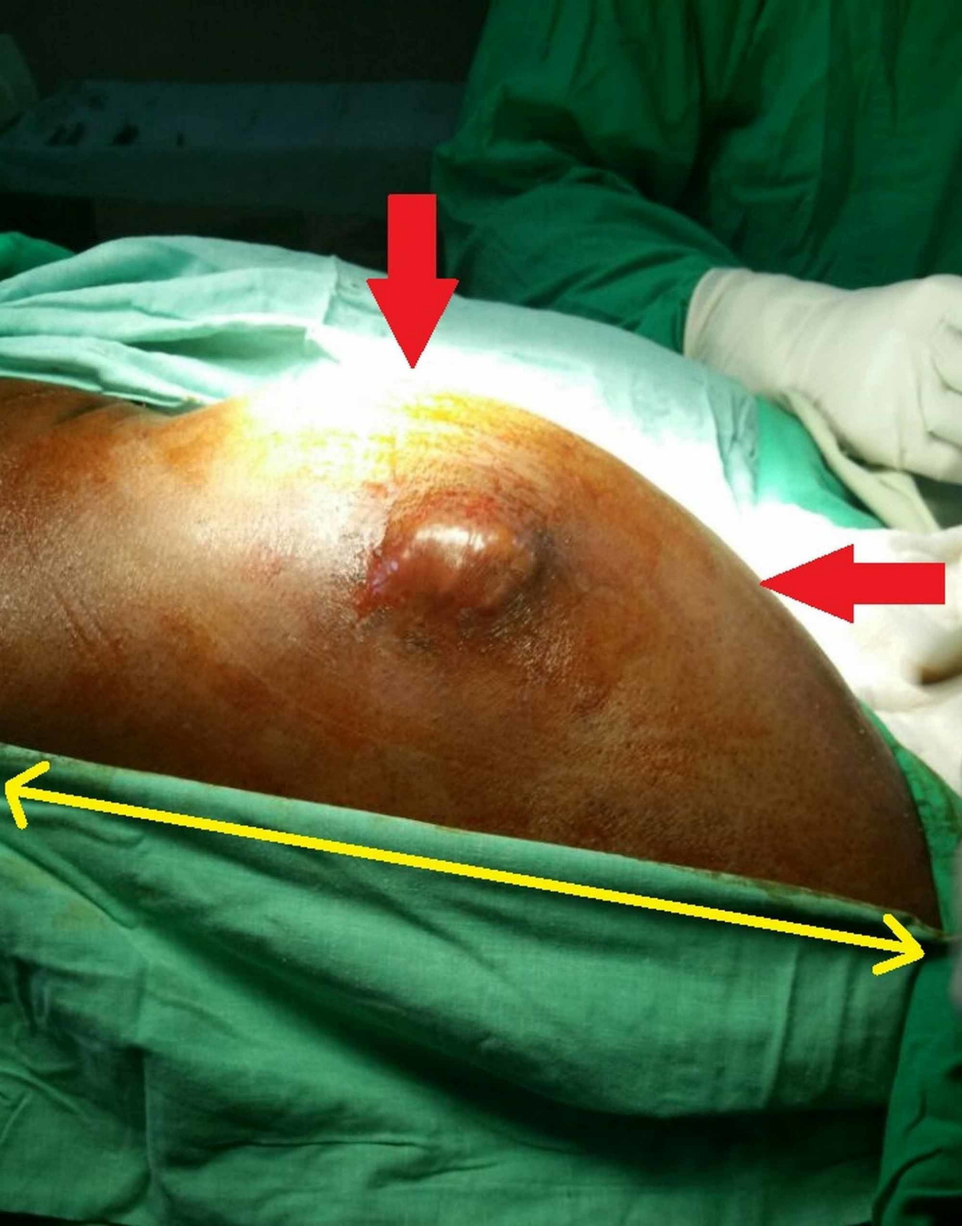
Preoperative appearance of the tumor on the patient’s left upper thigh (red arrows). The yellow arrow indicates the physical length of the tumor (30 cm).

Previous reports showed that two months back, the size of the swelling was 13×7 cm (length×width). On local examination, a well-defined mass measuring approximately 30×20 cm (length×width) was noted on the posteromedial aspect of the left upper thigh. Physical examination revealed that the swelling was warm, erythematous, firm in consistency, and non-compressible with a fluctuant center. The transillumination test was negative for the swelling. Figure 1 shows the preoperative appearance of the tumor.

Baseline investigations were conducted, which showed a low hemoglobin level (9.2 gm/dL) and raised total leukocyte count (18×10^3^/µL). The platelet count (388×10^3^/µL) was normal. His renal and liver function tests were unremarkable too. The detailed baseline investigations are summarized in Table 1.

**Table 1:** Baseline investigations of the patient, including complete blood count, renal, and liver function tests. MCV, mean corpuscular volume; BUN, blood urea nitrogen; ALT, alanine aminotransferase; ALP, alkaline phosphatase.

Investigations	Normal	Patient’s report	Comments
Complete blood count			
Hemoglobin	13-18 gm/dL	9.2	Decreased
Hematocrit	40%-50%	36	Decreased
MCV	80-100 fL	87.2	Normal
Total leukocyte count	4-11×10^3^/µL	18	Raised
Neutrophils	2.5-7.5×10^3^/µL	13.2	Raised
Lymphocytes	1.5-3.5×10^3^/µL	4.7	Raised
Platelets	150-450×10^3^/µL	388	Normal
Renal function tests			
BUN	6-20 mg/dL	19	Normal
Creatinine	0.7-1.2 mg/dL	0.9	Normal
Uric acid	3.4-7.0 mg/dL	5.2	Normal
Liver function tests			
Total bilirubin	<1.2 mg/dL	0.33	Normal
ALT	<50 U/L	45	Normal
ALP	50-136 U/L	87	Normal

**Figure 2:**
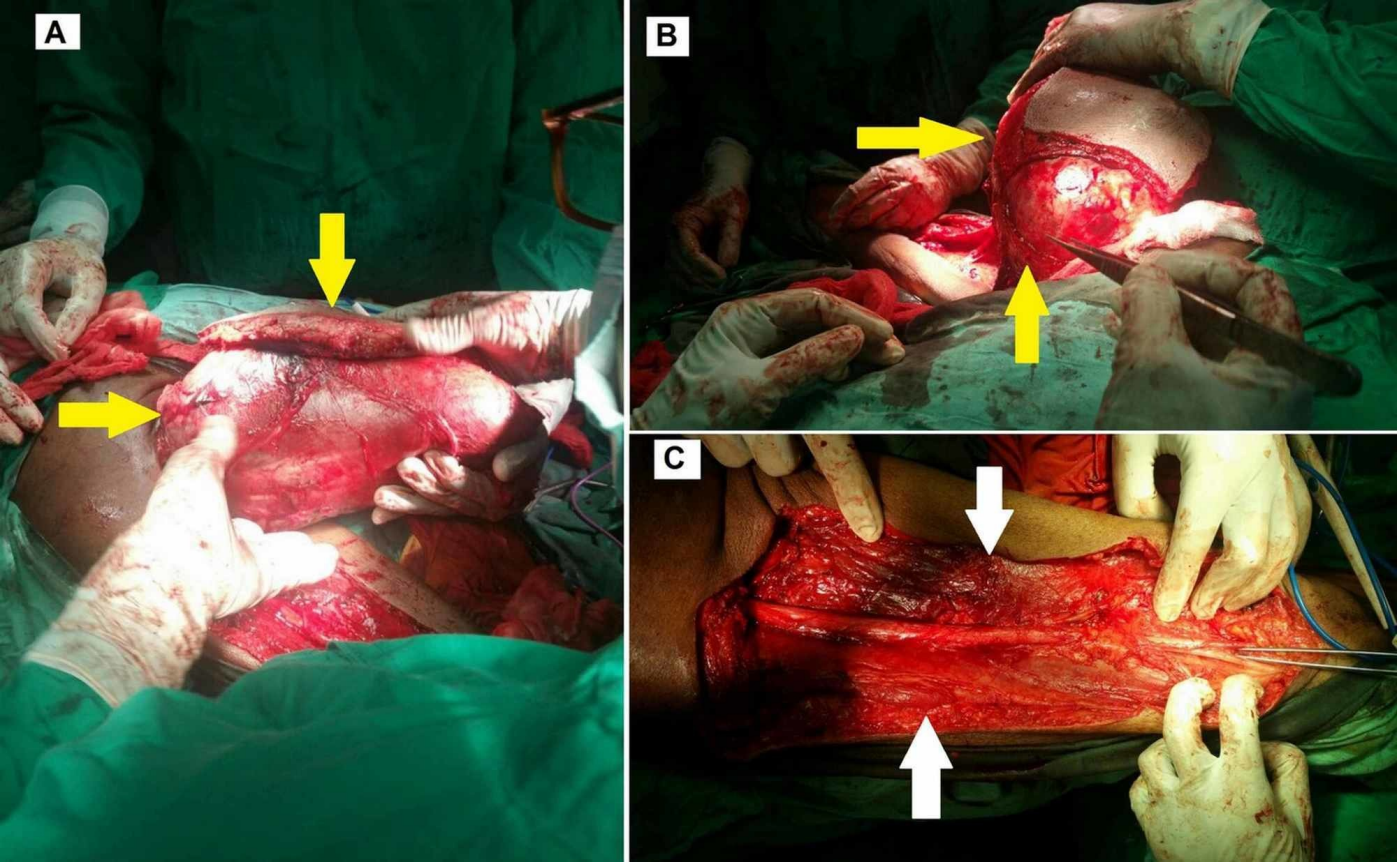
Perioperative images of leiomyosarcoma resection. Images A and B show the removal of firm encapsulated tumor (yellow arrows). Image C shows the posterior compartment of the left thigh (white arrows) after the removal of the tumor.

**Figure 3:**
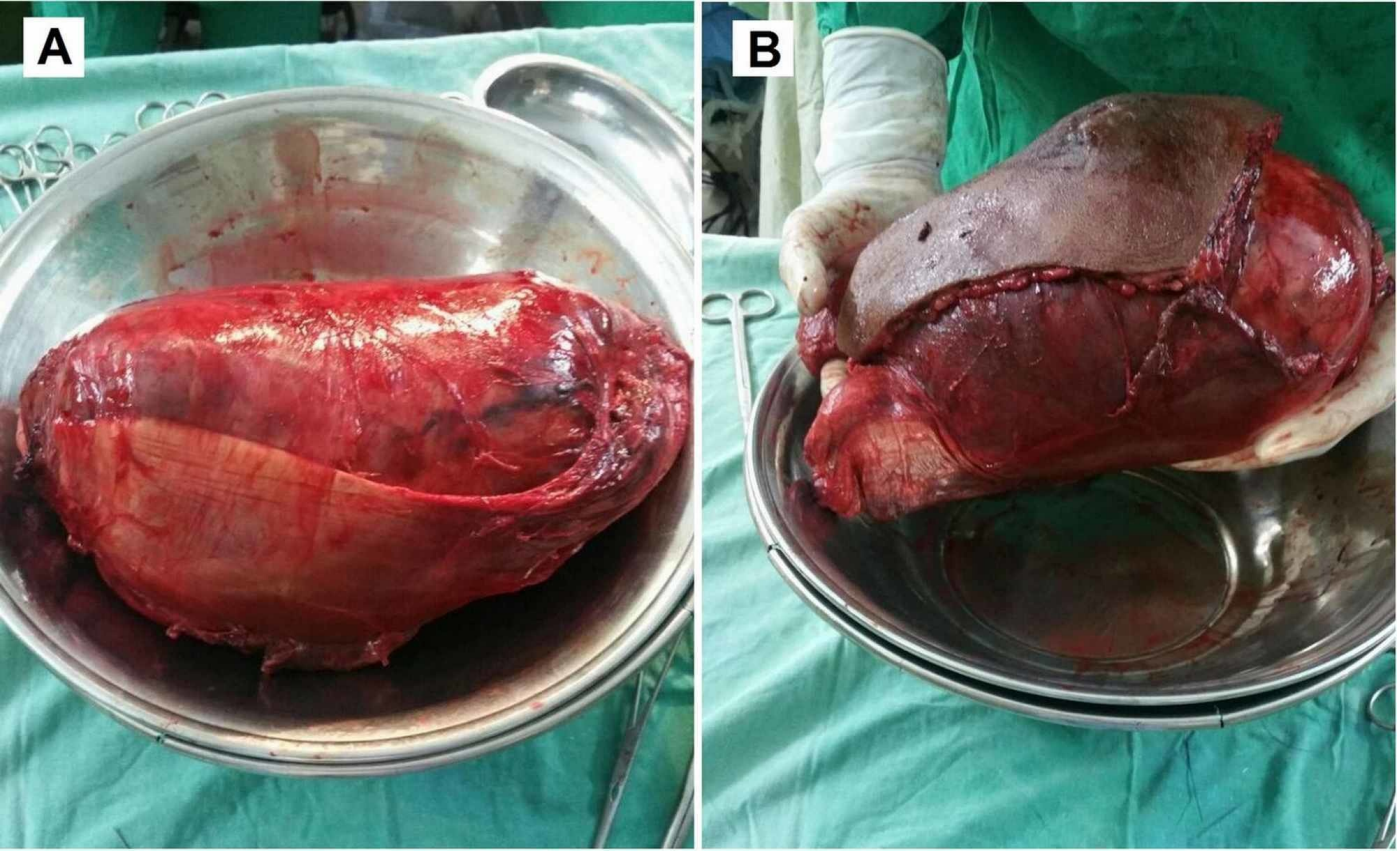
Gross appearance of excised leiomyosarcoma. Image A shows the gross morphology of the tumor measuring 24×17×9 cm in size. Image B depicts a comparison of the size of excised leiomyosarcoma relative to the surgeon’s hand.

**Figure 4:**
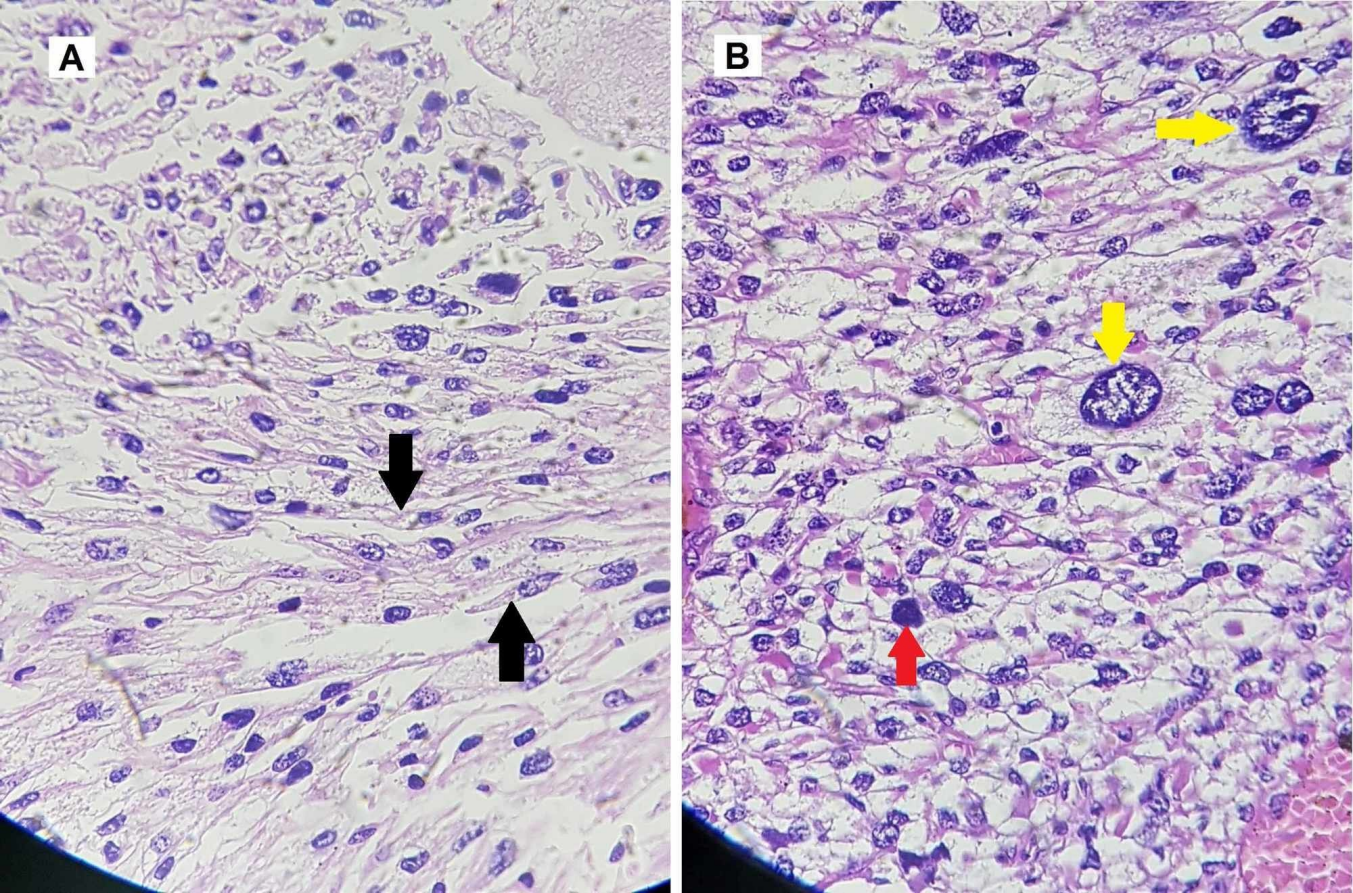
Histologic appearance of excised leiomyosarcoma. Image A shows spindle-shaped smooth muscle cells (black arrow). Image B is a magnified image showing mitotic figures (red arrow) and giant cells (yellow arrows).

Malignancy was suspected due to the patient’s history of weight loss and the rapidly increasing size of the mass. Imaging studies and necessary investigations were carried out. MRI showed that the mass involved the semitendinosus, semimembranosus, and biceps femoris muscles of the thigh. A biopsy was performed, and histopathology report showed multiple fragments of a neoplastic lesion. Immunohistochemical analysis confirmed poorly differentiated high-grade sarcoma, which was highly suggestive of leiomyosarcoma. The chest x-ray was normal and showed no evidence of pulmonary involvement. CT scan was done to look for metastasis; fortunately, no metastatic lesions were noted in the liver, lungs, and skeletal system. Surgery was planned to remove the mass. The patient underwent compartmental excision of the mass. Perioperative findings showed an encapsulated tumor in the biceps femoris, invading the fascia and skin over the posterior thigh (Figure 2). The final histopathology report showed a single, firm, brown, nodular tissue measuring 24×17×9 cm (length×width×height). The tumor cut surface was brown and hemorrhagic with nodular and cystic cavities. Histology revealed a sarcoma with moderate to marked nuclear pleomorphism, spindle-shaped smooth muscle cells, and a mitotic count of 20-25/10 high power field (HPF). The sample tested positive for alpha-smooth muscle actin (ASMA). Extensive autolytic and necrotic changes with multiple atypical mitotic figures and multinucleated tumor giant cells were seen on the cut sections. Figure 3 shows the gross appearance of the tumor on resection, and Figure 4 shows the microscopic and histologic appearance of the excised tumor.

**Figure 5:**
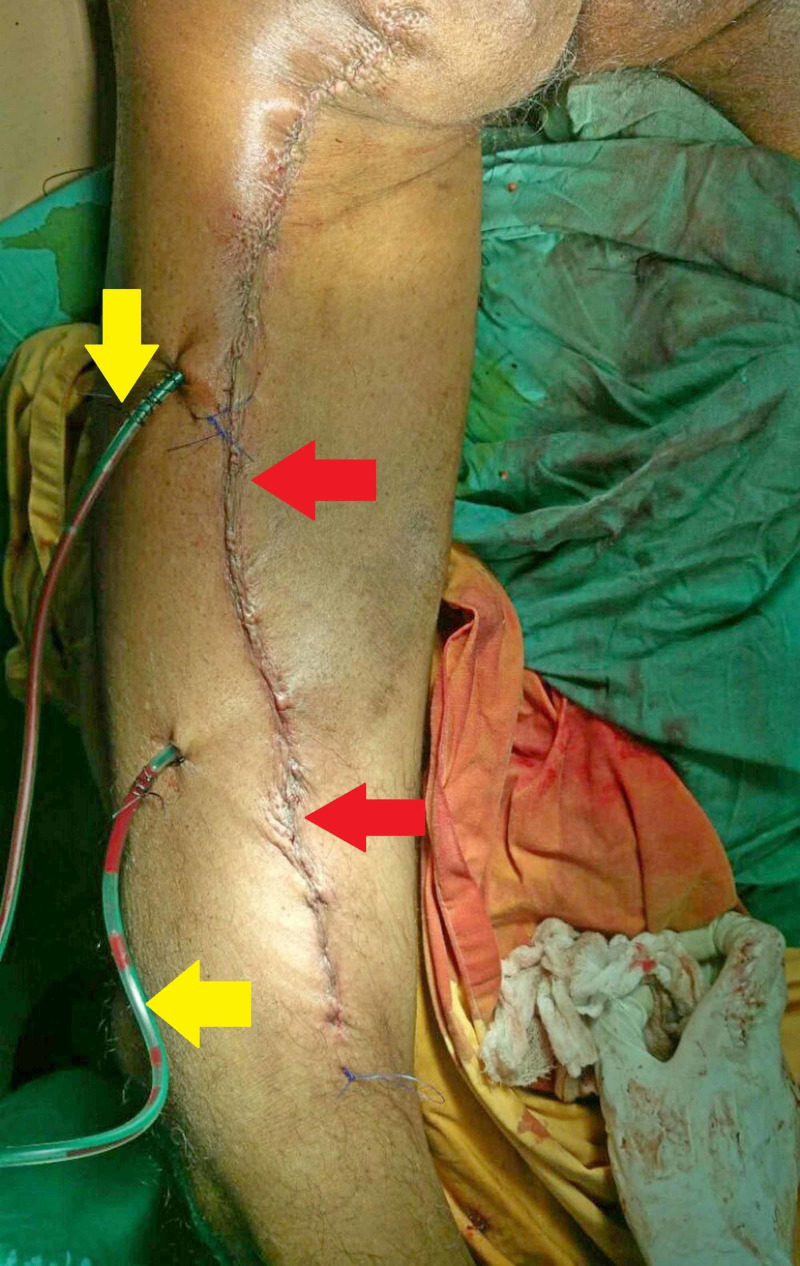
Patient's leg after resection of tumor. The red arrows show suture closure line after tumor resection surgery. The yellow arrows show drains placed at excision site.

Based on the histopathological findings, a final diagnosis of grade III leiomyosarcoma was made. The grading was done according to the Fédération Nationale des Centres de Lutte Contre Le Cancer (FNCLCC) grading of soft tissue sarcomas [10]. After resection, the surgical site was closed with sutures and drains were placed to avoid the collection of blood and fluid. Figure 5 shows the condition of patient’s leg after surgery. Postoperative recovery was normal, without any complications, and the patient was discharged after five days. He was referred to a rehabilitation clinic where he was followed up for two months as he regained the function of his legs. 

## Discussion

Leiomyosarcoma is a very rare malignancy. The development of this tumor outside of its usual cutaneous, uterine, retroperitoneal, and intra-abdominal regions is termed as ‘leiomyosarcoma of somatic soft tissues’ (SSTs). Cases of leiomyosarcoma of SSTs are poorly documented and not well studied due to their extremely low prevalence. Farshid et al., in their study, stated that leiomyosarcoma of SSTs has vascular origin [11]. The small population study conducted by him and his colleagues concluded that larger tumors of this type are more likely to disrupt and metastasize. Another study found that the size of the tumor is significantly correlated to recurrence [12]. As evidenced by our patient, leiomyosarcomas can be extremely fast-growing and locally aggressive, and hence the mass must be promptly excised once its nature has been confirmed via a biopsy.

The etiology of this type of cancer is unknown, and the risk factors have not been identified yet. The symptoms vary according to the location and stage of cancer, but common symptoms of cancer such as fatigue, fever, weight loss, malaise, nausea, and vomiting, are often seen. Pain in the area is uncommon, but swelling or mass is often noticed as the tumor is usually very fast growing [1,4-6,13]. Large tumors, especially in lower limbs, can affect the mobility of patients, as was seen in our case.

A primary diagnosis of the tumor can be made using basic imaging techniques, such as ultrasound, CT scan, and MRI [4,6,14]. The final diagnosis is confirmed after the histology has been studied. Spindle cell proliferation forming rough bundles and fascicles is seen under the microscope. The cells have cigar-shaped nuclei, and cytologic atypia and mitotic figures are also seen [14]. Our histologic findings (mentioned above) were similar to other cases of leiomyosarcoma reported in the literature [5,8,13,15]. The grading of leiomyosarcoma is done in accordance with FNCLCC guidelines. Three entities are considered for grading of sarcoma, namely tumor differentiation, mitotic index, and tumor necrosis. Each of these entities is given a score, and the total score defines the grade of sarcoma [10].

**Figure 6:**
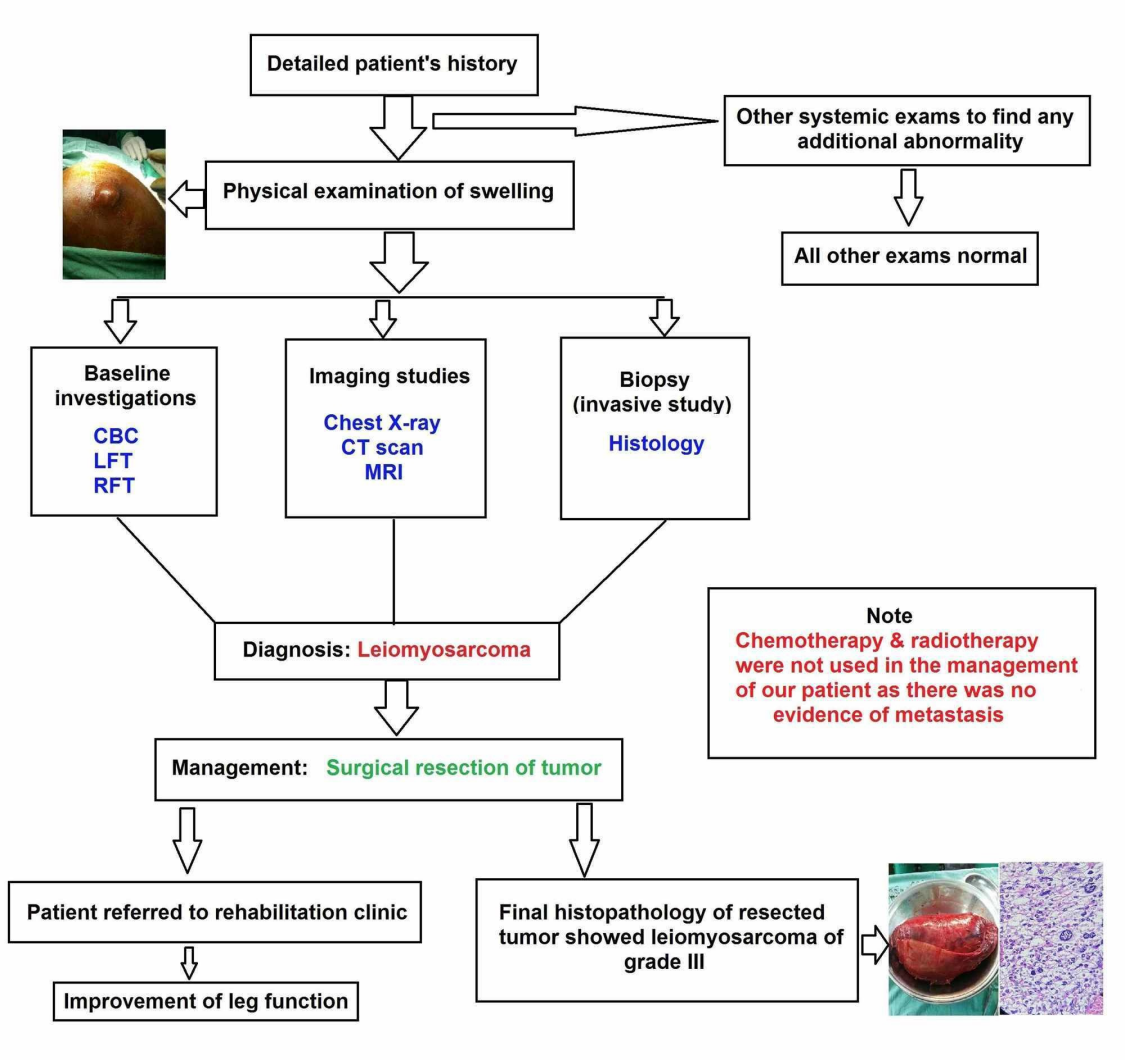
Summary of management of our patient. CBC, complete blood count; LFT, liver function test; RFT, renal function test.

The most common treatment is surgical resection, followed by radiation to eradicate any residual disease. Chemotherapy is used in the case of metastasis or recurrent disease [1,5,6]. The patients with soft tissue leiomyosarcomas have only a 50% chance of three-year survival, which makes this tumor very aggressive among soft tissue tumors [2]. After surgical removal of the tumor, our patient was referred to the rehabilitation clinic to improve his leg function. The summary of management of our patient is shown in Figure 6.

## Conclusions

Leiomyosarcomas can originate in many unusual locations. Only after biopsy and histology, the final diagnosis of rare malignancies can be made. Very few cases of leiomyosarcoma in thigh muscle have been previously reported; therefore, there are no guidelines related to the treatment protocols following surgery. Because of the increasing size and aggressive nature of the tumor, the need for rehabilitation and physical therapy of extremities should also be evaluated. The definitive treatment for leiomyosarcoma is surgery with wide margins removal. The need for chemotherapy and radiation, as for other leiomyosarcomas, needs to be evaluated in tumors arising in the limbs through trials. We conclude that clinicians should keep leiomyosarcoma as a differential when patients present with a rapidly growing mass in lower limbs. Early diagnosis and definitive treatment can improve the outcome of the patient and reduce the chances of metastasis, thus increasing survival. 
